# Revised manuscript R2, clean version are serum levels of 25-hydroxy vitamin D reduced following orchiectomy in testicular cancer patients?

**DOI:** 10.1186/s12610-021-00132-w

**Published:** 2021-06-10

**Authors:** Klaus-Peter Dieckmann, Osama Andura, Uwe Pichlmeier, Klaus Martin Otte, Hendrik Isbarn, Christian Wülfing

**Affiliations:** 1 Department of Urology, Testis Cancer Unit, Asklepios Klinik Altona, Paul Ehrlich Strasse 1, Hamburg, Germany; 2 Institut für Medizinische Biometrie und Statistik, Zentrum für Experimentelle Medizin, Universitätsklinikum Eppendorf, Hamburg, Germany; 3 Medilys Laborgesellschaft mbH, Hamburg, Germany; 4 Universitätsklinikum Eppendorf, Martini Klinik, Hamburg, Germany

**Keywords:** Testis, Germ cell tumor, Vitamin D, Orchiectomy, Hypovitaminosis, 25(OH)-Vitamin D, Testicules, Tumeur à Cellules Germinales, Vitamine D, Orchiectomie, Hypovitaminosis, 25(OH)-Vitamine D

## Abstract

**Background:**

The testis represents one place where the progenitor of vitamin D is converted into its active form. Loss of one testis was suggested to result in reduced vitamin D serum levels. Vitamin D deficiency would represent a significant health problem in the long-term course of patients with testicular germ cell tumors (GCTs) since most of them survive. The purpose of this study was to look to the serum 25(OH)-Vitamin D (25OHD) levels in patients with GCTs before and after orchiectomy. A total of 177 GCT patients underwent measurements of serum 25OHD levels, thereof 83 with preoperative measurements and 94 with measurements at six particular time-points from immediate postoperatively to >24 months. Longitudinal assessments of 25OHD serum levels were performed in individual patients with repeated measurements. A second analysis involved patient cohorts with measurements at six postoperative time-points. Serum levels of patients were also compared with 2 control groups, one consisting of 84 patients with non-neoplastic testicular diseases and another with 237 patients with non-neoplastic urologic diseases. We also looked to associations of 25OHD levels with levels of testosterone, follicle stimulating hormone (FSH), age, histology of GCT and season. Descriptive statistical methods were employed to compare groups and to analyze changes over time.

**Results:**

Normal serum levels of 25OHD were found in 21.7%, 23.1%, 20.2%, 21.9% in GCT patients preoperatively, after >2 years, in control group 1 and control group 2, respectively. Levels were significantly higher in spring and summer, but no association was found with other parameters. We found a significant transient decrease of 25OHD levels with a nadir at 6-12 months after orchiectomy and a recovery thereafter.

**Conclusion:**

Contrasting with previous studies we found no permanent reduction of serum 25OHD levels after orchiectomy but transient postoperative drop of 25OHD levels. There were no associations of 25OHD levels with age, and levels of testosterone or FSH. Our results may point to a particular role of the testis in vitamin D metabolism and may thus enhance the understanding of the diverse physiological roles of the testis.

## Résumé

**Contexte:**

Le testicule représente un lieu où le précurseur de la vitamine D est converti en sa forme active. Il a été suggéré que la perte d’un testicule pouvait induire une réduction des taux sériques de vitamine D. La carence en vitamine D représenterait un problème de santé important à long terme pour les patients présentant des tumeurs testiculaires à cellules germinales (TCG) puisque la plupart d’entre eux survivent. Le but de cette étude était de se tourner vers les taux sériques de 25(OH)-Vitamine D (25OHD) chez les patients présentant des TCG, avant et après orchidectomie. Au total, 177 patients avec TCG ont subi des mesures des taux sériques de 25OHD, dont 83 avec mesures préopératoires et 94 avec des mesures à six points de temps particuliers, de l’immédiat postopératoire jusqu’à plus de 24 mois. Des évaluations longitudinales des taux sériques de 25OHD ont été réalisées individuellement chez les patients avec des mesures répétées. Une seconde analyse a impliqué des cohortes de patients avec des mesures à six points de temps postopératoires. Les taux sériques des patients ont également été comparés à ceux de 2 groupes témoins, l’un composé de 84 patients atteints de maladies testiculaires non néoplasiques, et l’autre de 237 patients atteints de maladies urologiques non néoplasiques. Nous nous sommes également penchés sur les associations des taux de 25OHD avec les taux de testostérone, d’hormone folliculostimulante (FSH), l’âge, l’histologie de la TCG et la saison. Des méthodes statistiques descriptives ont été utilisées pour comparer les groupes et analyser les changements au fil du temps.

**Résultats:**

Des taux sériques normaux de 25OHD ont été trouvés chez 21,7%, 23,1%, 20,2%, et 21,9% des patients avec GCT, respectivement en préopératoire et après >2 ans, ainsi que chez les patients du groupe témoin 1 et du groupe témoin 2. Les taux ont été significativement plus élevés au printemps et en été, mais aucune association n’a été observée avec d’autres paramètres. Nous avons retrouvé une diminution transitoire significative des taux de 25OHD avec un nadir à 6-12 mois après orchiectomie et un rétablissement par la suite.

**Conclusion:**

Contrairement aux études précédentes, nous n’avons trouvé aucune réduction permanente des niveaux de sérum 25OHD après orchiectomie, mais une baisse postopératoire transitoire des taux de 25OHD. Il n’y avait pas d’associations entre les taux de 25OHD et l’âge, ni avec les taux de testostérone ou de FSH. Nos résultats peuvent mettre en avant un rôle particulier du testicule dans le métabolisme de la vitamine D et peuvent ainsi améliorer la compréhension des divers rôles physiologiques des testicules.

## Introduction

Testicular germ cell tumors (GCTs) represent one of the most frequent malignancies in men aged younger than 50 years [1]. The over-all cure rate is more than 90 % and most of the patients with GCT will enjoy a long life-expectancy despite the diagnosis of cancer [2]. Therefore, physicians caring for GCT patients are increasingly looking for long-term consequences of treatment, mainly second malignant neoplasms, cardiovascular disease and endocrinological disorders [3–5]. Recently, dysregulation of the vitamin D metabolism secondary to cancer-related loss of one testicle has come into the focus [6]. As vitamin D plays an essential role in bone health and in a large variety of other metabolic pathways [7–11] vitamin D deficiency would constitute a significant health problem in long-term survivors of GCT patients that care-givers of these patients should be aware of [12].

Physiologically, the progenitor of vitamin D, cholecalciferol, needs two hydroxylation steps to convert into its active form, the 1α, 25-dihydroxy vitamin D. The first hydroxylation step takes place in the liver and is mediated by CYP2R1, while the second hydroxylation is performed in the kidney under guidance of CYP27B1 [7–9, 13, 14]. Noteworthy, both enzymes CYP2R1 and CYP27B1, are found in large amounts in testicular tissue [15]. Immunohistologically, the enzymes are located in both germ cells and Leydig cells, and notably, reduced expression had been noted in men with impaired spermatogenesis [16, 17]. Furthermore, vitamin D receptors have been detected in germ cells and Sertoli cells [18, 19]. In animal experiments it has been shown that hepatic synthesis of CYP2R1 increases after castration [20]. Finally, an association of vitamin D serum levels with testosterone levels have been observed in elderly men [21], and vitamin D levels are associated with semen quality [22]. In aggregate, there is accumulating evidence for the involvement of the testicular compartment in the metabolism of vitamin D [16, 23].

Clinically, reduced vitamin D serum levels in men with testicular diseases were first reported by Foresta et al. in 2010 [6, 8, 24]. Two subsequent studies confirmed vitamin D deficiency in long-term survivors of GCT [25, 26].

The goal of the present investigation was to evaluate 25-hydroxyvitamin D (25OHD) serum levels in GCT patients prior to orchiectomy and during the later course. Our study hypotheses were as follows: (1) Loss of testicular parenchyma due to surgery is associated with decreased 25OHD serum level, (2) loss of both testicles will cause even lower 25OHD levels, (3) 25OHD deficiency will increase with time interval from testicular loss, (4) impaired spermatogenesis evidenced by increased levels of follicle stimulating hormone (FSH) is associated with 25OHD deficiency. In addition, we looked to possible associations of 25OHD deficiency with histology of GCT, patient age, season of the year, and testosterone-levels.

## Materials and methods

### Patients, controls

During 2018–2019, all patients undergoing orchiectomy for testicular GCT underwent preoperative measurements of 25-hydroxy vitamin D (25OHD) serum levels as part of the routine laboratory work-up (“base-line” measurement, “time-point 0”). Measurements were also done shortly postoperatively (p/o; “time-point 1”), and again at follow-up visits after 3 months (time-point 2), 6, 12, 24, and more than 24 months (time-point 6), respectively. Repeat measurements in individual patients were accomplished only in a few patients because most of the patients had their follow-up examinations after orchiectomy done elsewhere. Therefore, GCT patients who had orchiectomy performed at other institutions or before 2018 were enrolled for postoperative measurements of 25OHD serum levels with specifying the particular time-point after surgery. Some of whom underwent also repeat measurements.

As one of our study hypotheses was that orchiectomy-related 25OHD deficiency will increase with time from surgery, we defined a subgroup of patients where only the latest measurement available in each patient was employed for analysis. This subgroup is characterized as the “GCT main group”.

Controls consisted of patients with non-neoplastic scrotal diseases, e.g. epididymitis, varicocele, spermatocele, or hydrocele (“cohort 2”). A second control group (“cohort 3”) consisted of male urologic patients with non-testicular and non-neoplastic diseases, mainly urinary tract infection, stone disease, benign prostatic disease, and benign penile disease. All of the controls had only one measurement of 25OHD serum levels.

The following data were registered in GCT patients along with the serum level of 25OHD: time-point of measurement (time-points 0–6), histology of testicular tumor (seminoma or nonseminoma), season (1: January-March; 2: April-June; 3: July- September; 4: October – December), and serum levels of testosterone and follicle stimulating hormone (FSH) which always were measured as part of the routine care of GCT patients. In controls, no additional data were recorded.

All patients had given informed consent. Enquiry at the local ethical committee (Ärztekammer Hamburg, PV7288) revealed that no formal ethical approval was required because only anonymized data generated upon routine clinical care were analyzed. All study activities had been conducted according to the Declaration of Helsinki of the World Medical Association (as amended by the 64th General Assembly, 2013).

### Laboratory technology

Circulating serum 25OHD levels were measured by chemiluminescence micro-particle immunoassay (CMIA) on the Abbott Architect system. According to the serum levels measured, we defined the following categories of 25OHD levels: > 30ng/ml: normal; 20–30 ng/ml: insufficient (S1); 10–20 ng/ml: inadequate (S2); <10 ng/ml: deficient (S3).

### Evaluation of 25-hydroxyvitamin D serum levels in the various patient populations

We compared GCT-patients (GCT main group) with controls (Cohorts 2 and 3) quantitatively and qualitatively by analyzing proportions of serum level categories of 25OHD. To look for the hypothesized greater reduction of 25OHD levels in patients with bilateral tumors, we compared the measurements in patients with unilateral GCT to those of patients with bilateral tumors. For this comparison, we only used the measurements at the latest point of time available in the individual patients (GCT main group).

To look for decreases of 25OHD levels subsequent to orchiectomy, we compared preoperative levels (measurement 0) with those measured immediately after surgery (measurement 1) and also with the levels measured at follow-up visits (time-points 2–6). In a first step, we analysed intra-individual changes by including only patients with serial measurements available. For this purpose, we analyzed the median serum levels of the cohorts relating to the postoperative time-points and we also analyzed the frequencies of patients with decreasing and non-changing levels. However, as only few patients were available with more than 3 measurements and particularly with measurements beyond time-point 4, we performed a second analysis by comparing the cohort results available at the six postoperative time-points of measurements with regard to median serum levels and proportions of serum level categories.

In an exploratory approach, we looked to correlations of the 25OHD serum level in the GCT-main group with various parameters. For analysis of testosterone serum levels, we used the following categories of testosterone serum levels: subnormal (< 2.4 ng/ml), low-normal (2.4–3.2 ng/ml); normal (3.2–8.7 ng/ml); supranormal (> 8.7 ng/ml). For analysis of FSH-levels we used the following categories: low < 2 U/l; normal 2.0–11.9 U/l; slightly increased 12.0–15 U/l; highly increased > 15 U/l. To evaluate seasonal influences, the median and mean 25OHD levels in the seasons of the year were compared to each other. To look for associations of 25OHD levels with age, we compared the median serum levels of the following age-categories: <20 years; 20–29 years; 30–39 years; 40–49 years; ≥50 years. To look for associations with histology of testicular tumors, we compared the median serum levels of patients with pure seminoma to those with nonseminoma. This latter comparison was done with the baseline measurements and again with the GCT main group.

### Statistical methods

Minimum, 5 % percentile (P5), median, quartiles (Q1, Q3), 95 % percentile (P95) and maximum, interquartile ranges (IQR) and mean (with standard deviation, SD, and 95 % confidence limits) were calculated for the 25OHD levels in subgroups of GCT patients at the various time points of measurements and in controls. For each subgroup, we also calculated the relative proportions of the 25OHD serum level categories normal, and S1-S3. Results were documented as box-plot images whenever reasonable. For comparisons of proportions, the chi square tests was used. For comparison of two groups the Mann Whitney -U-test was applied and for more than two groups the Kruskal Wallis test. To increase statistical power by taking into account the correlation between repeated measurements within subjects, the Wilcoxon test and Friedman test were applied to compare two visits and more than two visits, respectively. To test any directional change of 25OHD level categories over time the Jonckhere Terpstra test was applied. P values were explorative, based on a significance level of 0.05. SAS software (version 9.4) was used for all statistical analyses.

## Results

### General results

A total of 501 men had measurements of 25OHD serum levels, thereof 177 patients with GCT (cohort 1; 112 seminoma, 65 nonseminoma), 87 patients with other testicular diseases (cohort 2) and 237 patients with non-testicular, non-neoplastic diseases (cohort 3). Details are given in Table 1. Nine patients had bilateral testicular tumors thereof six with serum levels measured postoperatively (p/o). Preoperative measurements were available in 83 GCT patients, 94 GCT patients were recruited at later points of time with many of whom undergoing repeated measurements. The patient samples at time-points 1 (immediately p/o) – 6 (> 24 months p/o) comprised of 84, 78, 39, 26, 24, and 26 measurements, respectively. The GCT main group consisted of 165 patients with 34 (20.6 %), 50 (30.3 %), 27 (16.4 %), 12 (7.2 %), 16 (9.7 %), 26 (15.8 %) having their last measurements at the time-points 1–6, respectively. In this group, the median measurement was at time point 2 (IQR 2–5).

**Table 1:** Characteristics of study population

	N	Age median (years)	Q1 (years)	Q3 (years)	min; max (years)
GCT patients	177	38	33;	46	19; 80
Seminoma	112	39	34.5	48.5	19; 80
Nonseminoma	65	35	29	41	19; 62
preoperative subgroup	83	38	33	47	19; 80
GCT main group	165	38	32	47	19; 80
Cohort 2	84	45	36	59	17; 82
Cohort 3	237	66	53	76	18; 94

cohort 2 denotes control group consisting of patients with non-neoplastic scrotal diseases; cohort 3 denotes control group 2 consisting of male urologic patients with non-testicular and non-neoplastic diseases

*Q1* first quartile, *Q3* third quartile

### Comparison of 25-hydroxy vitamin serum levels of GCT patients with controls

**Fig 1:**
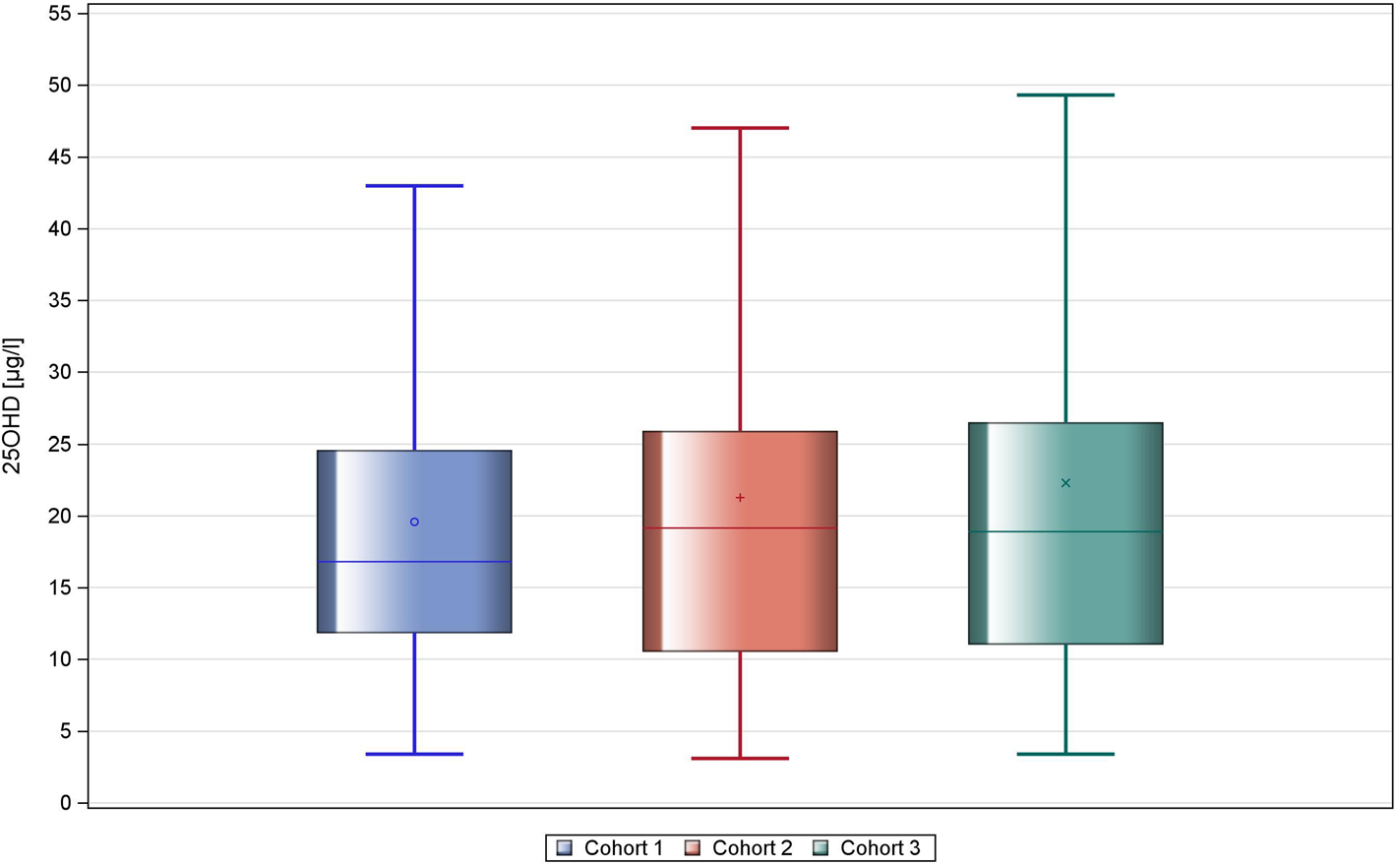
Comparison of serum vitamin D levels of GCT main group with controls. There is no significant difference of median 25OHD levels between the groups. Cohort 1 represents the GCT main group, cohort 2 denotes the first control group with nonneoplastic scrotal diseases. Cohort 3 represents the second control group that comprises of male urologic patients with nontesticular and non-malignant diseases

Prior to surgery, 21.7 % of GCT patients were found to have normal serum levels of 25OHD which is not different from the findings in cohort 2 (20.2 %) and cohort 3 (21.9 %), respectively. The frequencies of 25OHD serum level categories of the GCT main group and the two control groups are listed in Table 2. Statistical comparison did not reveal any significant difference (Kruskal Wallis test *p* = 0.1615). The calculated median and mean serum levels of the GCT main group and controls are listed in Table 3, and again, no significant differences were found between patients and controls (Fig. 1). Pooling of the two control groups did not change the result (*p* = 0.706 Mann Whitney U test, data not shown).

**Table 2:** 25OHD serum levels (ng/ml) in GCT main group and in controls: frequencies of categories of 25OHD serum levels

	Eligible (n)	Normal (n) (%)	20 – 30 ng/ml (n) (%)	10 – 19 ng/ml (n) (%)	<10 ng/ml (n) (%)	*p*-value^a^
GCT main group	165	25 (15.15%)	35 (21.21%)	68 (41.21%)	37 (22.42%)	
Cohort 2	84	17 (20.2%)	22 (26.2%)	26 (31.0%)	19 (22.6%)	
Cohort 3	237	52 (21.9%)	57 (24.1%)	83 (35.0%)	45 (19.0%)	
						0.1615

cohort 2 denotes control group consisting of patients with non-neoplastic scrotal diseases; cohort 3 denotes control group 2 consisting of male urologic patients with non-testicular and non-neoplastic diseases

^a^Kruskal Wallis Test

**Table 3:** 25OHD serum levels (ng/ml) in GCT main group and controls

	n	Min	Q1	Median	Q3	Max	Mean	SD
GCT main group	165	3.4	10.50	15.80	24.40	142	19.63	15.56
Cohort 2	84	3.1	10.50	19.15	25.90	85.1	21.27	14.54
Cohort 3	237	3.4	11.00	18.90	26.50	148.1	22.30	17.38

*median* median 25OHD serum levels, *mean*: mean 25OHD serum level values, *Q1* first quartile, *Q3* third quartile, *SD* standard deviation

cohort 2 denotes control group consisting of patients with non-neoplastic scrotal diseases; cohort 3 denotes control group 2 consisting of male urologic patients with non-testicular and non-neoplastic diseases

*P* = 0.1797 Kruskal Wallis Test for over-all comparison of GCT main group with the two other cohorts

### Comparison of unilateral GCT with bilateral GCT

The median serum 25OHD levels of patients with bilateral GCT and of those with unilateral disease are not significantly different. Details are given in Table 4.

**Table 4:** 25OHD serum levels (ng/ml) in patients with unilateral and bilateral GCT

	n	median	Q1; Q3	Min; max	mean	SD	*p*-value
Unilateral GCT^a^	159	15.80	10.50; 24.40	3.40; 142.40	19.62	15.63	
Bilateral GCT^a^	6	14.0	9.40; 27.30	7.70; 46.70	19.85	14.84	
							*P* = 0.8721^b^
Cohort 2	84	19.15	10.5; 25.9	3.1; 85.1	21.27	14.54	
Cohort 3	237	18.9	11.0; 26.5	3.4; 148.1	22.3	17.38	
							*P* = 0.1797^c^

*Q1* first quartile, *Q3* third quartile, *SD* standard deviation

cohort 2 denotes control group consisting of patients with non-neoplastic scrotal diseases; cohort 3 denotes control group 2 consisting of male urologic patients with non-testicular and non-neoplastic diseases

^a^ in each patient, the latest postoperative measurement value available was employed for analysis

^b^Mann Whitney U test for comparison of unilateral versus bilateral tumors

^c^Kruskal Wallis test for comparison of all cohorts

### Longitudinal changes of 25-hydroxy vitamin D levels

**Fig. 2:**
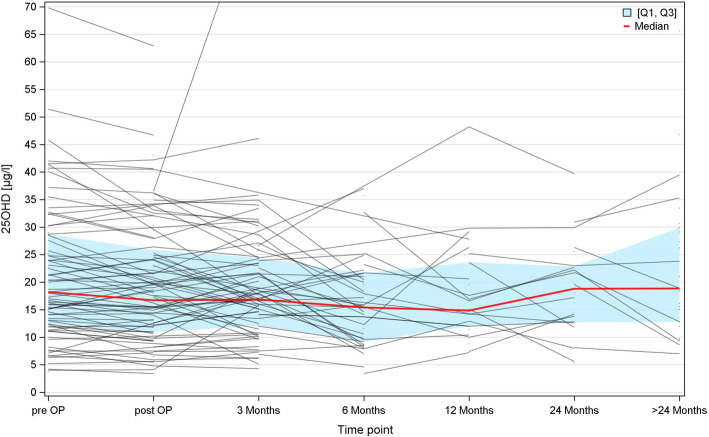
Longitudinal changes of 25OHD serum levels in individual patients. Each line represents one individual patient. The red line represents the median 25OHD level of all patients eligible at the various points of time. It shows a slight decline from baseline to the 6 months and 12 months time point and a subsequent increase thereafter. Q1 first quartile, Q3 third quartile

Of the patients who had serial measurements available, 64.7 % had decreased 25OHD levels 6 months after orchiectomy by comparison with baseline levels (further details in Table 5 and Fig. 2). Accordingly, there is a significant decrease of median serum 25OHD levels from baseline to time-point 3 (6 months p/o), both by pairwise comparing the median levels of baseline with the 6-months measurement (Wilcoxon test, *p* = 0.0497) and by analyzing the changes of the cohort course over time (Friedman test, *p* = 0.0122). Details are given in Table 6. Due to low numbers with complete sets of serial measurement at all time points, the serum levels at 12 months are not statistically different from baseline despite their even lower measurement results.

**Table 5:** Shift table: Comparing 25OHD baseline measurement values with measurements at each later time point – intra-individual analysis of complete cases only

Time point	n^a^	Decrease (n; %)	No change (n;%)	Increase (n;%)
Baseline	83	-	-	-
Immediate p/o	57	13 (22.8%)	43 (75.4%)	1 (1.8%)
3 months p/o	44	13 ( 29.6%)	20 ( 45.4%)	11 ( 25.0%)
6 months p/o	17	11 ( 64.7%)	2 ( 11.8%)	4 ( 23.5%)
12 months p/o	5	3 ( 60.0%)	1 ( 20.0%)	1 ( 20.0%)

Jonckhere-Terpstra test for change over time *p* = 0.0743. The last value (12 months p/o) was not included in statistical analysis because of too small numbers

*p/o* postoperatively

^a^only complete cases were included, i.e. those with serial measurements available

**Table 6:** Changes of 25OHD levels (ng/ml) over time. Intra-individual analysis of cases with serial measurements

Time point	Measures	Results per visit	Results at baseline	Results per visit	Changes from baseline
preoperative	n	83			
	Mean (SD)	21.21 (12.29)			
	Median (Q1, Q3)	18.20 (12.30, 28.70)			
Immediately p/o	n	84	57	57	57
	Mean (SD)	19.48 (11.58)	21.28 (13.22)	19.33 (12.08)	-1.95 (3.40)
	Median (Q1, Q3)	16.70 (10.95, 25.85)	17.00 (12.30, 27.60)	15.50 (10.90, 24.10)	-1.10 (-4.00, 0.10)
	*p*-value^a^				0.3258
3 mon p/o	n	78	44	44	44
	Mean (SD)	21.12 (18.22)	20.93 (10.73)	19.37 (9.33)	-1.56 (7.51)
	Median (Q1, Q3)	16.85 (12.10, 24.40)	18.45 (14.60, 28.15)	17.90 (12.00, 24.50)	0.70 (-6.95, 3.75)
	*p*-value^a^				0.5482
6 mon p/o	n	39	17	17	17
	Mean (SD)	16.28 (8.14)	23.79 (9.94)	16.58 (7.62)	-7.21 (10.90)
	Median (Q1, Q3)	15.40 (9.20, 21.60)	24.00 (16.00, 28.50)	15.90 (9.80, 20.40)	-8.20 (-15.20, 0.50)
	*p*-value^a^				0.0497
	*p*-value^b^				0.0122

*p/o* postoperatively, *mon* months, *SD* standard deviation, *Q1* first quartile, *Q3* third quartile

^a^ Wilcoxon-Test for the comparison of paired measures (baseline with post-OP, baseline with 3 Months and so on)

^b^ Friedman-Test for the comparison of the cohort course over time for all measures until 6 Months post-operative visit

If patients who had measurements available at particular time-points were grouped according to the particular time-points of measurements, no significant change over time was observed regarding the proportions of 25OHD serum level categories (Table 7) with regard to the entire period of observation (Jonckhere-Terpstra test, *p* = 0.1913). However, if the proportions of categories of time-point 3 (6 months p/o) are compared to the distribution of categories at baseline, there is a significant decrease of proportions with normal levels (Kruskal Wallis test, *p* = 0.0270) with only 7.7 % of patients having normal 25OHD serum levels at this point of time. Thereafter, there is a further slight decrease of the proportion of normal levels until the 12 months time-point followed by a recovery to normal after 2 years. Details are given in Table 7.

**Table 7:** Changes over time: frequencies of categories of 25OHD serum levels at different time points

Time-point	Eligible (n)	Normal (n) (%)	20 – 30 ng/ml (n) (%)	10 – 19 ng/ml (n) (%)	<10 ng/ml (n) (%)	*p*-value^a^	*p*-value^b^
0 (preoperatively)	83	18 (21.7%)	22 (26.5%)	30 (36.2%)	13 (15.7%)	-	-
1 (immediate p/o)	84	15 (17.9%)	19 (22.6%)	31 (36.9%)	19 (22.6%)	0.232	-
2 (3 months p/o)	78	12 (15.4%)	20 (25.6%)	35 (44.9%)	11 (14.1%)	0.4397	-
3 (6 months p/o)	39	3 (7.7%)	10 (25.6%)	14 (35.9%)	12 (30.8%)	0.0270	0.0737
4 (12 months p/o)	26	1 (3.9%)	7 (26.9%)	15 (57.7%)	3 (11.5%)	0.1605	0.0559
5 (24 months p/o)	24	2 (8.3%)	8 (33.3%)	10 (41.7%)	4 (16.7%)	0.3905	0.0793
6 (>24 months p/o)	26	6 (23.1%)	6 (23.1%)	9 (34.6%)	5 (19.2%)	0.8444	0.1913

*p/o* postoperatively

^a^Kruskal Wallis test for comparison of proportions at specific time-point with baseline

^b^Jonckhere-Terpstra test for hypothesis of decrease of serum levels over time until specific point of time

**Fig. 3:**
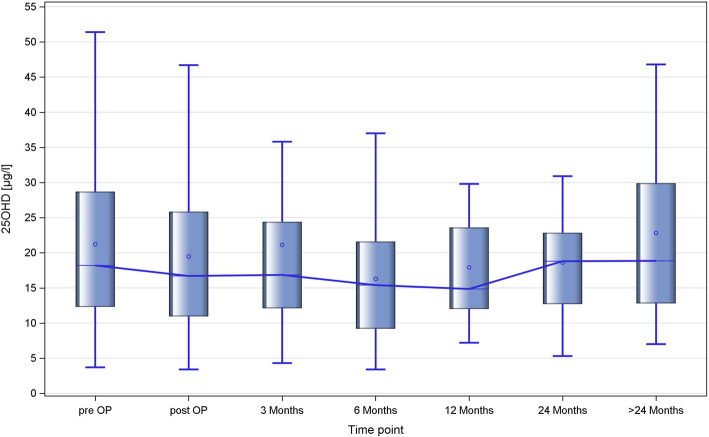
Longitudinal changes of serum 25OHD levels in GCT patients (box plot image). Each box denotes the median 25OHD serum levels with interquartile ranges in cohorts at consecutive time-points. The connecting line demonstrates a decrease of levels reaching a nadir at the 6 months time point and subsequent rise of levels thereafter. Preop: preoperative measurement, post-op measurement after orchiectomy

Table 8 and Fig. 3 provide measurement results of the patient cohorts with available 25OHD measurements at the various time-points. Again, the median and mean serum levels decrease after orchiectomy to reach a nadir at 6 months (mean) and 12 months (median) followed by an increase reaching even higher levels than preoperatively at the last time-point of measurement.

**Table 8:** Changes of 25OHD serum levels (ng/ml) over time: median and mean values at different time points

Time-point of Measurement	n	MIN	P5	Q1	MEDIAN	Q3	P95	MAX	MEAN	LCLM	UCLM
0	83	3.7	6.3	12.30	18.20	28.70	41.5	69.8	21.21	18.53	23.90
1	84	3.4	5.6	10.95	16.70	25.85	40.6	62.9	19.48	16.97	22.0
2	78	4.3	6.2	12.10	16.85	24.40	35.8	142.4	21.12	17.01	25.23
3	39	3.4	4.6	9.20	15.40	21.60	35.7	37.0	16.28	13.64	18.92
4	26	7.2	7.4	12.00	14.85	23.60	29.8	48.2	17.92	14.24	21.60
5	24	5.3	5.6	12.70	18.80	22.85	30.9	39.7	18.61	15.05	22.18
6	26	7.0	8.6	12.80	18.85	29.90	46.8	65.6	22.81	17.30	28.32

*P5* 5% percentile; Q1 first quartile; Q3 third quartile; *P95* 95% percentile; *LCLM* Lower 95% confidence limit of mean

*UCLM* upper 95% confidence limit of mean

### Results of exploratory search for associations

**Fig. 4:**
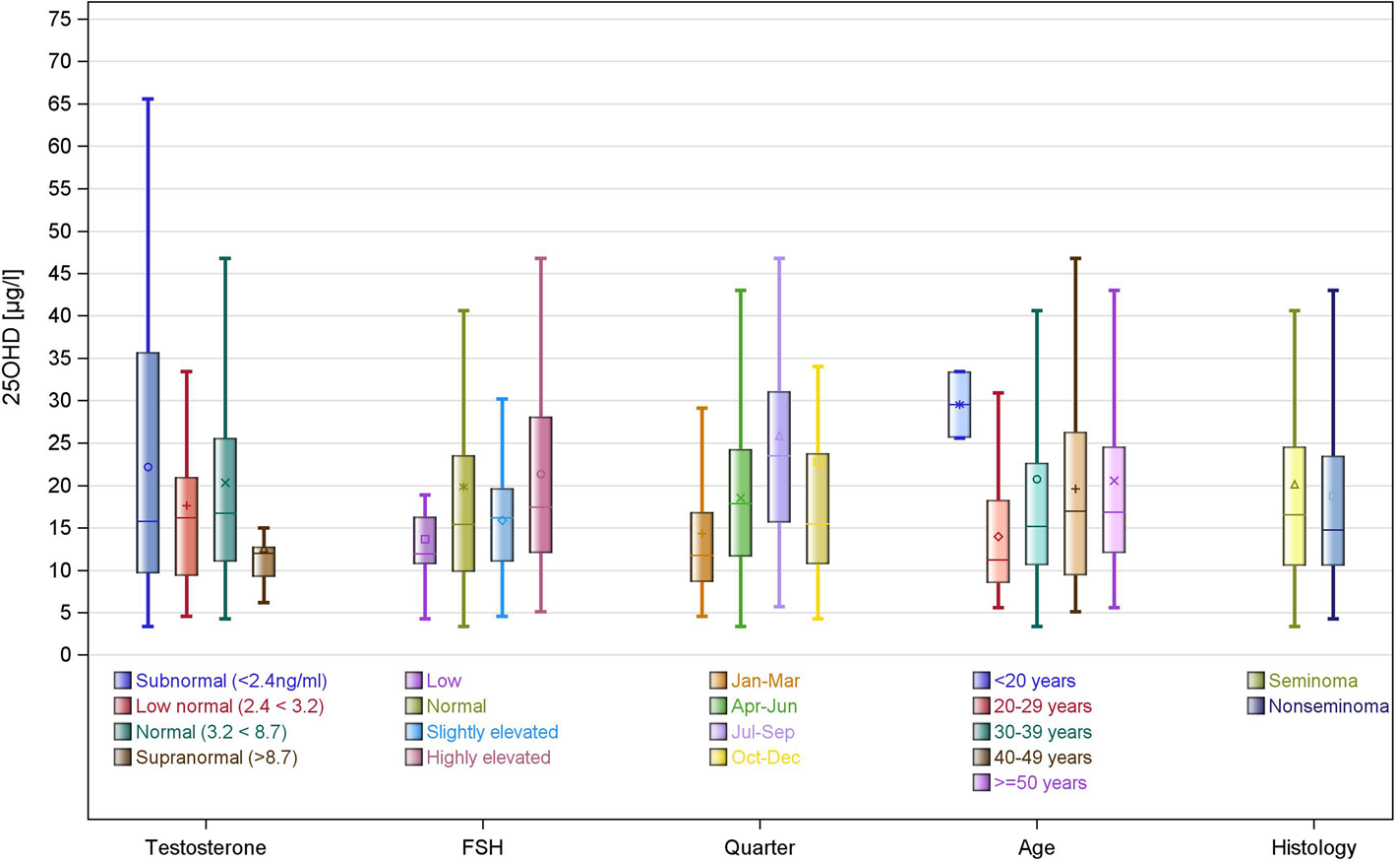
Possible associations of serum 25OHD levels with clinical factors. Box-plots of categories of serum levels of testosterone and FSH, of age, season, and histology of GCT. A significant association of 25OHD serum levels was only detected with quarter of the year. Ranges of FSH serum levels: low < 2 U/l; normal 2.0 – 11.9 U/l; slightly increased 12.0 – 15 U/l, highly increased >15 U/l

The results of the exploratory analysis of possible associations of 25OHD serum levels with various clinical parameters are listed in Tables 9, 10, 11, 12 and 13, and Fig. 4. Briefly, significantly higher 25OHD levels are observed in quarters 2 and 3 of the year (April to September,) than in the other seasons (Table 11, Fig. 4), whereas no significant associations were found with categories of testosterone levels, categories of FSH, age categories, and histologic subgroups of GCT (Kruskal Wallis test, all analyses p > 0.05).

**Table 9:** 25OHD levels in relation to serum testosterone (GCT main group)

		25OHD serum level (ng/ml)		
categories of testosterone levels (ng/ml)	n	Median (Q1;Q3)	Mean (SD)	*p*-value^a^
Subnormal (<2.4)	19	15.80 (9.60, 35.70)	22.23 (16.52)	
Low normal (2.4 – <3.2)	19	16.20 (9.30, 21.00	17.63 (9.49	
Normal (3.2 - <8.7)	114	16.75 (11.00, 25.60)	20.34 (16.78)	
Supranormal (>8.7)	13	12.00 (9.20, 12.80)	12.59 (5.92)	
				0.1566

*Q1* first quartile, *Q3* third quartile, *SD* standard deviation

^a^Kruskal Wallis test

**Table 10:** 25OHD levels in relation to Follicle Stimulating hormone (FSH) levels (GCT main group)

		25OHD serum levels (ng/ml)		
categories of FSH levels	n	Median (Q1;Q3)	Mean (SD)	*p*-value^a^
Low (<2 U/l)	8	11.95 (10.70, 16.35)	13.70 (6.81)	
normal (2 – 11.9 U/l)	88	15.45 (9.80, 23.60)	19.88 (18.39)	
Slightly elevated (12 – 15 U/l)	18	16.20 (11.00, 19.70)	15.89 (7.24)	
Highly elevated (>15 U/l)	50	17.45 (12.00, 28.10)	21.39 (13.05)	
				0.2644

*Q1* first quartile, *Q3* third quartile, *SD* standard deviation

^a^Kruskal Wallis test

**Table 11:** 25OHD levels in relation to quarter of the year (GCT main group)

		25OHD serum levels (ng/ml)		
categories	n	Median (Q1;Q3)	Mean (SD)	*p*-value^a^
Jan - Mar	58	11.80 (8.60, 16.90)	14.34 (8.23)	
Apr - Jun	34	17.90 (11.60, 24.30)	18.56 (9.05)	
Jul - Sep	37	23.50 (15.60, 31.10)	25.84 (14.93)	
Oct - Dec	36	15.50 (10.70, 23.80)	22.79 (24.87)	
				<.0001

*Q1* first quartile, *Q3* third quartile, *SD* standard deviation

^a^Kruskal Wallis test

**Table 12:** 25OHD levels in relation to patient´s age (GCT main group)

		25OHD serum levels (ng/ml)		
Age categories	n	Median (Q1; Q3)	Mean (SD)	*p*-value^a^
<20 yrs	2	29.50 (25.60, 33.40)	29.50 (5.52)	
20 – 29 yrs	22	11.25 (8.50, 18.30)	13.98 (7.59)	
30 – 39 yrs	67	15.20 (10.60, 22.70)	20.80 (20.42)	
40 – 49 yrs	45	17.00 (9.40, 26.30)	19.60 (10.95)	
>50 yrs	29	16.90 (12.00, 24.60)	20.59 (12.88)	
				0.0785

*Yrs* years

*Q1* first quartile, *Q3* third quartile, *SD* standard deviation

^a^Kruskal Wallis test

**Table 13:** 25OHD levels in relation to histology of primary tumor

		25OHD serum levels (ng/ml)		
Histologic subgroup	n	Median (Q1; Q3)	Mean (SD)	*p*-value^a^
Seminoma, lav	101	16.60 (10.50, 24.60)	20.18 (16.72)	
Nonseminoma, lav	64	14.80 (10.50, 23.55)	18.77 (13.60)	
				0.5306
Seminoma, baseline	60	18.45 (12.75, 28.70)	21.44 (12.53)	
Nonseminoma, baseline	23	18.00 (11.30, 27.60)	20.63 (11.87)	
				0.8547

lav Latest available value in individual patients

*Q1* first quartile, *Q3* third quartile, *SD* standard deviation

^a^Kruskal Wallis test

## Discussion

The central result of the present study is that no significant reduction of 25-hydroxyvitamin D serum levels could be detected in patients with testicular germ cell tumors neither at the time of diagnosis nor in the long-term course. However, there appears to be a transient reduction of 25OHD serum levels during the first year after orchiectomy followed by a recovery after 2 years. In GCT patients, 25OHD serum levels are associated with season, but no significant associations were found with testosterone levels, FSH levels, age and histology of the testicular neoplasm.

The presence of enzymes CYP2R1 and CYP27B1 and the detection of vitamin D receptor expression in testicular tissue as well as evidence for clinically relevant vitamin D deficiency in hypogonadal men [17, 23, 27–29] suggested a prominent role of the testis in the metabolism of this vitamin [15, 16, 19, 24]. Accordingly, three clinical studies reported higher incidences of subnormal 25OHD serum levels in patients orchiectomized for GCT particularly during long-term follow-up [24–26]. However, the results of the present investigation are clearly at variance with these studies. The reasons for the conflicting results are not clear, but sample sizes and methodological discrepancies could account for disconformities. The three previous studies comprised of rather small sample sizes of 15, 61, and 82 patients, respectively, whereas our GCT main group had as many as 165 patients. Thus, chance effects might have influenced the results of previous studies. Accordingly, the p-values in one of the studies were just slightly below the level of significance [25]. Furthermore, one study based the results only on the reference limits of the 25OHD serum test without a formal control group [26]. However, subnormal levels of 25OHD are observed in up to 40 % of the general population in western countries [30] which is even worse in the present study where we found normal levels of only 20.2 and 21.9 %, respectively, in the two control groups.

Foresta et al. suggested that total loss of testicular parenchyma by bilateral orchiectomy would result in a significant reduction of vitamin D metabolizing capacity and might thus cause decreased serum levels [6]. That hypothesis is quite appealing because it would be in line with the observation of low 25OHD levels in hypogonadal men [23], but it is based on only 15 patients. Our sample size is even smaller with respect to the subgroup of bilateral GCT, but we did not observe significantly different 25OHD serum levels among GCT patients with bilateral and unilateral disease and controls. No other study has addressed the vitamin D status in patients with total loss of testicular tissue so far. Therefore, no final conclusion can be drawn to date because the numbers of patients tested are clearly too small.

Although we did not observe a permanent reduction of 25OHD levels after orchiectomy, we noted a significant and continuous decrease postoperatively during the first year reaching a nadir between 6 and 12 months after surgery. Thereafter, the median levels as well as the proportion of normal levels raised again to reach levels equivalent to preoperative findings and to controls. This observation is unique so far since none of the previous studies with GCT patients had examined preoperative 25OHD levels and looked to these patients at subsequent visits. Our finding is statistically significant and it is methodologically sound because it was obtained in an analysis employing exclusively individual patients with serial measurements. The significant decrease was confirmed by an analysis using cohorts comprising of all patients with available measurements at the various time-points. These subgroups consisted of varying patient compositions. Thus, the significant decrease of 25OHD serum levels during the first year after orchiectomy is based on two methodological approaches. Yet, the results still rest on small sample sizes and clearly need further confirmation.

Our finding of a transient decrease of 25OHD serum levels subsequent to orchiectomy would be in line with the hypothesis that testicular tissue may play an important role in the metabolism of vitamin D. A probable explanation for the recovery of 25OHD levels after two years of time, would be that the testis is obviously not an essential site of vitamin D biosynthesis and other organs holding the hydroxylases CYP2R1 and CYP27B1 (e.g. liver, kidneys) will compensate the loss of testicular tissue after some time. This hypothesis would accord with animal experiments that had revealed an increased synthesis of CYP2R1 in the liver after loss of both testes [18, 20]. Another hypothetic explanation would be that the remaining testicle could increase its particular metabolic activity and might thus compensate the loss of one testis.

In our exploratory analysis of associations of parameters with 25OHD serum levels, we confirmed that significantly higher serum levels are found in April to September than in the first and fourth quarter. This result is completely in line with current knowledge [7, 9, 22], and it reflects the impact of more sun-light in spring and summer which is required for the very first step of vitamin D activation in the skin [8, 18]. Noteworthy, we found no association of 25OHD serum levels with testosterone serum levels although such an association had been suggested by investigations of infertile men [22, 23, 29, 31–33]. In GCT patients, one small study found an association of testosterone levels with 25OHD serum levels [25], but one other study reported conflicting results [26]. In view of the present data, the weight of evidence is probably in favor of a null association of 25OHD levels with testosterone status.

Limitations of the present studies relate to small patient numbers in several subgroups at different time-points despite the acceptable over-all sample size. Also, the varying compositions of the subgroups at the various time-points do certainly represent a weak-point. A confounding effect could result from different median ages of the GCT patients and the two control groups. However, that effect is probably low, because we did not note an association of age with 25OHD levels as reported in the exploratory search section. The seasonal variation of 25OHD serum levels might have influenced our results, particularly the findings of serum level changes over time. However, the decrease of 25OHD levels was observed after an interval of 6–12 months in individual patients and seasons had changed several times during the individual observation periods. Also, seasonal variation is present in both patients and controls and as all subjects were concurrently recruited during the two years study period, this factor should not considerably affect the comparisons of patients with controls. The effects of additional treatment procedures were not accounted for. However, as additional procedures had been applied mostly in nonseminomas and as there was no significant difference between seminomas and nonseminomas, the confounding effect of additional treatment is probably low. Finally, the majority of patients were evaluated during the first two years after orchiectomy. Thus, our data do not allow for a clear conclusion regarding the developments of 25OHD levels in a truly long-term course.

## Conclusions

The hypothesis of a vitamin D deficiency in survivors of testicular GCT is not supported by the results of this study. However, we detected a transient decrease of 25OHD serum levels during the first year after orchiectomy with a recovery after 2 years. Thus, a minor role of the testis in the metabolism of vitamin D seems still conceivable, but obviously, loss of testicular tissue does not necessarily result in clinically relevant vitamin D deficiency.

## Data Availability

The datasets used and/or analyzed during the current study are available from the corresponding author on reasonable request.

## Abbreviations

GCT: germ cell tumor
25OHD: 25-hydroxy vitamin D
FSH: follicle stimulating hormone
p/o: postoperatively
IQR: interquartile range
SD: standard deviation
