# Correction to: Are serum levels of 25-hydroxy vitamin D reduced following orchiectomy in testicular cancer patients?

**DOI:** 10.1186/s12610-021-00136-6

**Published:** 2021-06-28

**Authors:** Klaus-Peter Dieckmann, Osama Andura, Uwe Pichlmeier, Klaus Martin Otte, Hendrik Isbarn, Christian Wülfing

**Affiliations:** 1 Department of Urology, Testis Cancer Unit, Asklepios Klinik Altona, Paul Ehrlich Strasse 1, Hamburg, Germany; 2 Institut für Medizinische Biometrie und Statistik, Zentrum für Experimentelle Medizin, Universitätsklinikum Eppendorf, Hamburg, Germany; 3 Medilys Laborgesellschaft mbH, Hamburg, Germany; 4 Universitätsklinikum Eppendorf, Martini Klinik, Hamburg, Germany

**Correction to: Basic Clin Androl 31, 14 (2021)**

**https://doi.org/10.1186/s12610-021-00132-w**

Following publication of the original article [1], an error was identified in the article title due to a typesetting mistake.

The incorrect article title read: Revised manuscript R2, clean version are serum levels of 25-hydroxy vitamin D reduced following orchiectomy in testicular cancer patients?

The correct article title is: Are serum levels of 25-hydroxy vitamin D reduced following orchiectomy in testicular cancer patients?

The article title has been updated above and in the original article. The publisher apologises to the authors and readers for the inconvenience caused by this mistake.
